# Tiagabine induced modulation of oscillatory connectivity and activity match PET-derived, canonical GABA-A receptor distributions

**DOI:** 10.1016/j.euroneuro.2021.04.005

**Published:** 2021-09

**Authors:** Alexander D. Shaw, Hannah L. Chandler, Khalid Hamandi, Suresh D. Muthukumaraswamy, Alexander Hammers, Krish D. Singh

**Affiliations:** a Cardiff University Brain Research Imaging Centre (CUBRIC), School of Psychology, Cardiff University, CF24 4HQ, Wales; b School of Pharmacy, Faculty of Medical and Health Sciences, University of Auckland, Auckland, New Zealand; c King's College London & Guy's and St Thomas’ PET Centre, School of Biomedical Engineering and Imaging Sciences, St Thomas' Hospital, London SE1 7EH, United States

**Keywords:** Neuropharmacology, MEG-PET, Receptor-mapping

## Abstract

As the most abundant inhibitory neurotransmitter in the mammalian brain, γ-aminobutyric acid (GABA) plays a crucial role in shaping the frequency and amplitude of oscillations, which suggests a role for GABA in shaping the topography of functional connectivity and activity. This study explored the effects of pharmacologically blocking the reuptake of GABA (increasing local concentrations) using the GABA transporter 1 (GAT1) blocker, tiagabine (15 mg). In a placebo-controlled crossover design, we collected resting magnetoencephalography (MEG) recordings from 15 healthy individuals prior to, and at 1-, 3- and 5- hours post, administration of tiagabine and placebo. We quantified whole brain activity and functional connectivity in discrete frequency bands. Drug-by-session (2 × 4) analysis of variance in connectivity revealed interaction and main effects. Post-hoc permutation testing of each post-drug recording vs. respective pre-drug baseline revealed consistent reductions of a bilateral occipital network spanning theta, alpha and beta frequencies, across 1- 3- and 5- hour recordings following tiagabine only. The same analysis applied to activity revealed significant increases across frontal regions, coupled with reductions in posterior regions, across delta, theta, alpha and beta frequencies. Crucially, the spatial distribution of tiagabine-induced changes overlap with group-averaged maps of the distribution of GABA_A_ receptors, from flumazenil (FMZ-V_T_) PET, demonstrating a link between GABA availability, GABA_A_ receptor distribution, and low-frequency network oscillations. Our results indicate that the relationship between PET receptor distributions and MEG effects warrants further exploration, since elucidating the nature of this relationship may uncover electrophysiologically-derived maps of oscillatory activity as sensitive, time-resolved, and targeted receptor-mapping tools for pharmacological imaging.

## 1 Introduction

There is a pressing need to develop time-resolved non-invasive markers of pharmacological effects within the brain, that can be used to both drive our understanding of brain function but also demonstrate the action and temporal dynamics of novel pharmacological agents. This could help reduce the very significant costs associated with the development of new drugs, by helping to demonstrate direct target engagement, and therefore accelerate the development of novel treatments for diseases that have significant societal burdens. One strong candidate for such a marker are neural oscillations, either modulated by task or characterised at rest. Unlike indirect functional magnetic resonance imaging (fMRI), neural oscillations measured using non-invasive methods, such as magnetoencephalography (MEG), arise directly from the synchronous oscillatory-activation of post-synaptic currents in ensembles of principal cells in cortex (Brunel and Wang, 2003; Buzsáki et al., 2004; Wang, 2010), predominantly those in superficial layers (Buzsáki et al., 2012; Xing et al., 2012). Converging evidence from experimental (Kramer et al., 2008; Whittington et al., 1995) and computational (Brunel and Wang, 2003; Pinotsis et al., 2013; Shaw et al., 2017) studies suggests that the attributes of these oscillations – peak frequency and amplitude – are determined by more fundamental, unobserved, neurophysiological processes. For example, the peak frequency of oscillations across many frequency bands has been linked with inhibitory neurotransmission (Buzsáki et al., 2004; Hall et al., 2011; Roopun et al., 2006; Whittington et al., 2000) mediated by γ-aminobutyric acid (GABA).

The link between GABA and macroscopic oscillations observed in MEG is likely mediated by the functional inhibitory role of GABA receptors, which are broadly categorised as fast, ionotropic GABA_A_ and slower G-protein coupled GABA_B_, although there exist subtypes of each (Belelli et al., 2009; Vargas, 2018; Whiting, 2003). The currents mediated by both of these receptor types have been implicated in oscillation generation through control of recurrent excitation-inhibition (Atallah and Scanziani, 2009; Brunel and Wang, 2003; Galarreta and Hestrin, 1998; Kohl and Paulsen, 2010; Krupa et al., 2014), whereby the GABAergically-mediated inhibitory currents exert temporal control over the firing and activity of excitatory principal cells (Bartos et al., 2007; Whittington et al., 2000). In the case of higher-frequency oscillations, including beta (13 - 30 Hz) and gamma (30+ Hz), the peak frequency may be dependant on the balance of excitatory and inhibitory currents (Brunel and Wang, 2003; Kujala et al., 2015; Shaw et al., 2017), implicating excitatory glutamatergic receptor types, such as α-amino-3‑hydroxy-5-methyl-4-isoxazolepropionic acid (AMPA) and N-methyl-D-aspartate (NMDA).

Pharmaco-MEG studies provide a framework for examining the effects that pharmacological manipulation of neurotransmitter or neuromodulator systems has on oscillatory activity. To date, studies of GABAergic drugs have primarily examined changes in task-induced fluctuations in oscillation metrics, usually confined to the analysis of one particular sensory system. For example, in the motor cortex, benzodiazepines, such as diazepam, modulate the amplitude of movement-related beta oscillations (Hall et al., 2011; Jensen et al., 2005). In visual cortex, propofol, an agonist of GABA_A_ receptors, increased the amplitude of gamma oscillations and concurrently suppressed the amplitude of alpha oscillations (Saxena et al., 2013). Tiagabine reduced the frequency of stimulus-induced gamma oscillations (Magazzini et al., 2016). Alcohol, which has a complex binding profile including benzodiazepine-mediated GABA_A_ effects, decreased the frequency and increased the amplitude of gamma in the visual cortex (Campbell et al., 2014). For a summary of GABAergic pharmaco-MEG studies, see (Muthukumaraswamy, 2014; Nutt et al., 2015). These results clearly demonstrate a role for GABA in shaping oscillatory responses, however they are confined to observations of task-related, local dynamics.

Task-free, ‘resting state’ recordings of oscillations focus on correlation of fluctuations in different brain regions, within specific frequency bands. This functional connectivity approach rests on the assumption that discrete regions of the brain whose band-limited amplitude envelopes are correlated, are functionally connected. Since GABA plays a role in shaping the oscillations of local (within-region) dynamics, it is expected that it also shapes the topography of the networks formed by these regions interacting.

In the present study, we examined the effect of pharmacologically blocking the reuptake of GABA using tiagabine, a GABA-transporter 1 (GAT1) blocker. We anticipated increased GABA availability would increase activation of GABAergic receptors and thereby GABAergic inhibition, which would have a macroscopic effect of reducing functional connectivity strengths across frequency bands.

Next, we examined the degree to which these changes overlap with the spatial distribution of GABA_A_ receptors in a group-average map of flumazenil volume-of-distribution (FMZ-V_T_) PET. Here, we expected brain regions showing the highest FMZ-V_T_ (interpreted as density of GABA_A_ receptors) would also demonstrate the biggest drug-induced changes in oscillatory activity.

## 2 Experimental procedures

### 2.1 MEG methods

#### 2.1.1 Sample

Fifteen healthy individuals (1 female, 14 male) aged between 20 and 32 (mean 25.5) completed a single-blind, placebo-controlled crossover study of 15 mg oral tiagabine (Gabitril) and placebo. Study days were separated by at least a week, with each day consisting of 4 MEG scans; an initial ‘pre-drug’ recording, followed by post-ingestion scans at 1, 3 and 5 h. MEG data from the active task protocols, including an overview of the design, has been published previously (Magazzini et al., 2016; Muthukumaraswamy et al., 2013). All study participants gave informed consent and the study procedures were approved by the UK National Research Ethics Service (South East Wales). Volunteers were excluded if they reported personal history of psychiatric or neurological diagnosis, current recreational or prescription drug use, or impaired liver function. Participants completed the Subject High Assessment Scale (SHAS) (Schuckit, 1980) immediately following the end of each scan.

#### 2.1.2 Data acquisition

Whole head MEG recordings were obtained using a CTF275 radial gradiometer system, with participants seated upright. Task-free, ‘resting’ recordings of 10 min were obtained at 1200 Hz and analysed as synthetic third-order gradiometers (Vrba, 2001). Participants were fitted with three electromagnetic head coils (nasion and bilateral pre-auriculars), which remained fixed throughout the study day, and were localized relative to the MEG system immediately before and after recording. All study participants had a T1-weighted MRI (1 mm isotropic) available for subsequent source-space analysis. Co-registration was achieved by placement of fiduciary markers at fixed distances from anatomical landmarks easily identifiable on an anatomical MRI (tragus, eye centre). Offline, recordings were downsampled to 600 Hz and segmented into 2 s epochs. Each epoch was visually inspected for gross artefact (e.g. movement, clenching, eye blinks) and removed from the analysis.

#### 2.1.3 Resting-state analysis pipeline

**Fig. 1:**
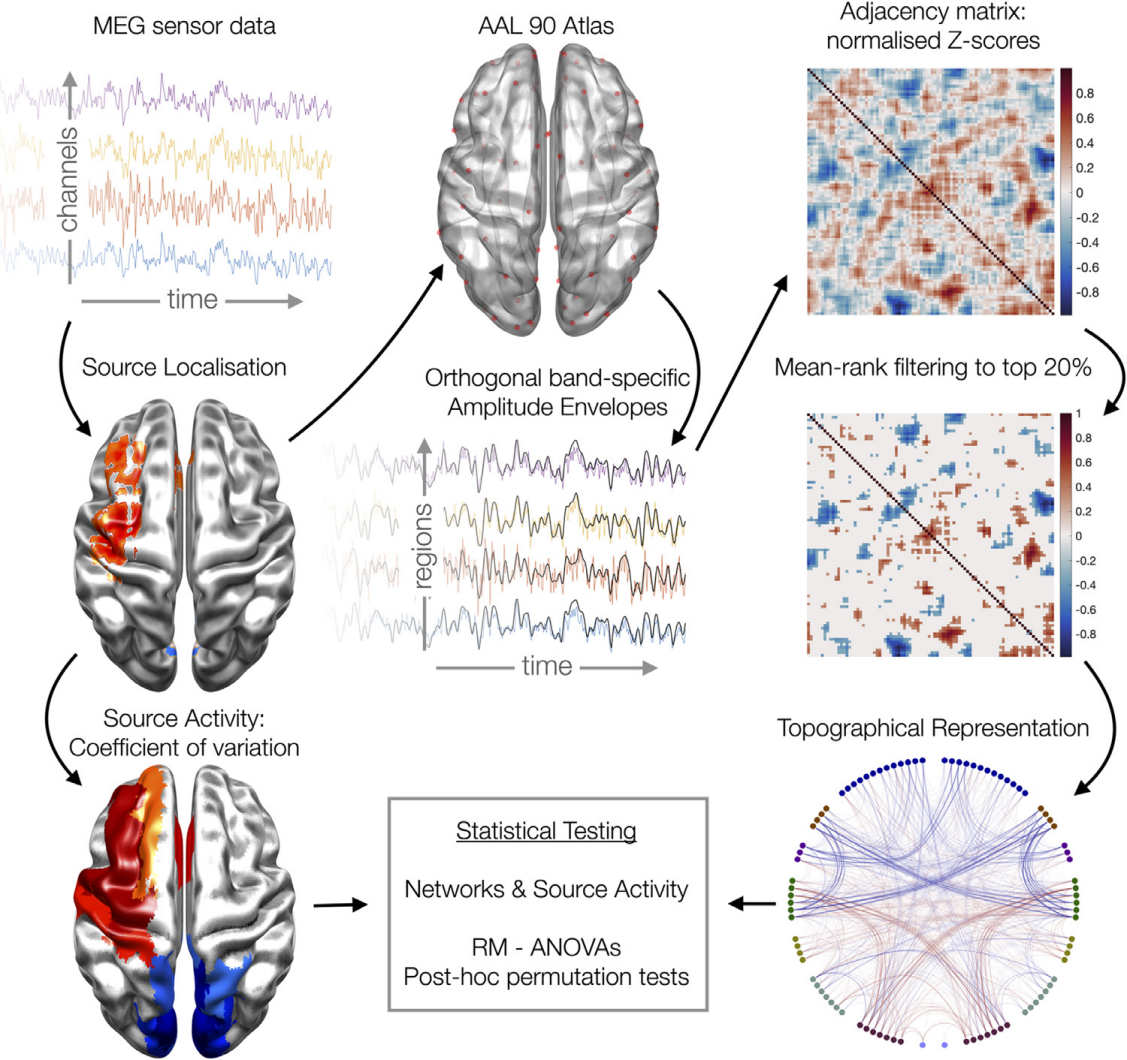
Schematic overview of the analysis and statistical pipeline. RM-ANOVA: repeated measures analysis of variance.

We computed functional connectivity using the amplitude envelope correlation (AEC) metric, which is interpretable, robust and repeatable (Colclough et al., 2016). This coupling was computed separately for 7 distinct frequency bands using conventional definitions; delta (1 – 4 Hz), theta (4 – 8 Hz), alpha (8 – 13 Hz), beta (13 - 30 Hz) and 3 gamma windows (40 – 60, 60 – 80 and 80 – 100 Hz). Fig. 1 summarises the analysis pipeline.

Source analysis was performed using the linearly constrained minimum-variance (LCMV) beamformer, a spatial filtering method, as implemented in Fieldtrip (Oostenveld et al., 2011). Using a local-spheres conductivity (forward) model, the beamformer estimated the temporal activity of each location of a 6 mm grid, as linear combinations of the MEG channels. This step was repeated for each of the frequency bands of interest, with the covariance of the MEG channels being recomputed from appropriately band-pass filtered data. For each of the locations on this grid, the estimated source timeseries for each trial was concatenated to form a single timeseries for both connectivity and activity analysis.

#### 2.1.4 Analysis of amplitude-amplitude connectivity

Our analysis proceeded using the methods we employed in Koelewijin et al. (2019). We first reduced the spatial dimensionality of the source reconstructed data to a set of 90 anatomical loci, described by the Automatic Anatomical Labelling (AAL90) atlas (Tzourio-Mazoyer et al., 2002). We chose one grid source to represent each AAL90 region, selected based on the voxel having the largest temporal standard deviation across the resting-state experiment.

Having reduced to a set of 90 regions, the temporal activity of sources was orthogonalized with respect to each other region using symmetric orthogonalization (Colclough et al., 2015), which further reduces source leakage. Next, the amplitude envelopes of each region were extracted using the absolute of the (complex) analytical signal derived by Hilbert transform (in MATLAB). This timeseries was downsampled to a temporal resolution of 1 s in order to study connectivity mediated by slow amplitude envelope changes (Brookes et al., 2011). A median filter was used to remove spikes or transients within the envelope.

Functional connectivity of the 90 regions was calculated using cross-correlations of the amplitude envelopes of each region. Since correlations are undirected, only the upper triangular portion (minus the diagonals) of the 90-by-90 correlation matrix was computed, requiring 4005 computations for each of the frequency bands tested, for each subject. In order to make these correlations more suitable for statistical analysis, and to correct for the varying length of the final timeseries for each person, each of these correlation coefficients were then transformed to variance-normalised Fisher z-statistics, using a procedure that estimates the effective temporal variance of the node timeseries’ null distribution using surrogates generated by randomisation.

We additionally adjusted each individual connectivity matrix to correct for global session effects (Siems et al., 2016). These effects can be generated by experimental session confounds effecting SNR, such as head-size, head-motion and position within the MEG helmet. Such correction procedures are common in fMRI connectivity analyses, although there is much debate as to the optimum algorithm to be used for post-hoc standardization (Yan et al., 2013). Here we adopted a variant of z-scoring, in which the null mean and standard-deviation of connectivity, across the matrix, is estimated by fitting a Gaussian (Lowe et al., 1998) to the noise peak (+/- 1SD) of the distribution. This estimated mean and SD is used to Z-score each Fisher's Z connectivity value for that person/session.

Finally, in order to only analyse connections which are strongly present in every person/session, we estimated the mean rank of every connection in the matrix and removed the weakest 80%.

#### 2.1.5 Source activity analysis

For each of the beamformer voxels on the 6 × 6 × 6 mm reconstruction grid (5061 voxels), we estimated source activity for each voxel, frequency, person and session. Estimating raw power estimates from MEG/EEG data, in source-space, and comparing these across people and sessions is problematic because geometrical effects, which vary from session to session and across the brain, can lead to artefactual inter-session differences (Luckhoo et al., 2014). This is particularly important for beamformer reconstructions in which typical corrections for depth-biases in single-state filter weights can exacerbate these artefactual differences. Here, we use a normalised measure of “activity”, in which we estimate the amplitude envelope at each of the 5061 locations, using the Hilbert methods described above, and calculate the temporal coefficient of variation (CoV) i.e. the temporal standard deviation of the envelope, divided by the mean of the envelope. Finally, we also z-scored these activity measures using the same gaussian-fit procedure described above for our connectivity measures.

### 2.2 PET flumazenil methods

#### 2.2.1 Sample

A group of 16 healthy participants (females: 4, age range: 26–61 years, mean age: 46 years) were recruited as healthy controls for a clinical PET study. No participants in the study reported history of neuropsychiatric or neurological conditions or were on any prescribed medications. None of the participants consumed alcohol within 48 h prior to their PET-FMZ scan. All participants provided written informed consent according to the Declaration of Helsinki. Approvals from the UK Administration of Radioactive Substances Advisory Committee (ARSAC) and the Ethics Committee, Imperial College, Hammersmith Hospital, were obtained. For the creation of the FMZ template, data from six healthy participants with global values close to the mean were selected and raw and right-left reversed (flipped) data were used (12 scans).

#### 2.2.2 PET scan parameters and image analysis

Dynamic 3D PET data were collected on a 953B Siemens/CTI PET scanner. Data consisted of 20 frames over a 90-minute time period. Scans were obtained with axial planes parallel to a horizontal plane passing through the anterior and posterior commissures. The tracer, ~370 MBq of [^11^C]FMZ, was injected intravenously and arterial blood samples were collected in order to calculate the plasma input function with metabolite correction (Hammers et al., 2003; Lammertsma et al., 1993).

Voxelwise parametric maps of [^11^C]FMZ total volume-of-distribution (V_T_) were calculated from the time-series data and arterial plasma input functions with spectral analysis (Cunningham and Jones, 1993; Lassen et al., 1995), allowing for a blood volume fraction, as detailed previously (Hammers et al., 2008).

#### 2.2.3 Template creation

Statistical Parametric Mapping software was used to create the [^11^C]FMZ PET template (SPM99, Wellcome Trust Centre for Human Neuroimaging, University College London, London, UK, https://www.fil.ion.ucl.ac.uk/spm/software/spm99/).

PET data were coregistered to the corresponding MRI data, and left-right-reversed, but not resliced. The MRIs were normalised to the Montreal Neurological Institute standard space (MNI152). The PETs were spatially normalised using the same transformations as for the MRI images and written with voxel sizes of 2 × 2 × 2 mm. Data were then averaged with a softmean function and smoothed with an 8 mm (FWHM) Gaussian kernel to yield the final template.

### 2.2.4 Statistical analyses

For connectivity and activity analyses, we employed a repeated-measures analysis of variance (RM-ANOVA), of drug (tiagabine, placebo) by session (pre and 1-, 3- and 5-hours post). Where drug x session interaction effects were present, we employed post-hoc paired-t testing to explore how effects evolved dynamically across sessions (time).

For connectivity analyses, computed in AAL90 space for computational tractability, we employed randomisation based post-hoc testing with omnibus-correction (Nichols and Holmes, 2001). For the activity analysis, performed at the voxel level (‘voxelwise’), permutation testing was not computationally tractable; as such we employed false-discovery-rate (FDR) correction (Benjamini and Hochberg, 1995). For the comparison of the spatial distribution of activity effects with the PET FMZ V_T_ template, we used randomisation-based Pearson's correlation (5000 permutations) with omnibus correction.

## 3 Results

### 3.1 Functional connectivity

Supplementary Fig. 1 (S1) shows connections (edges) demonstrating significant drug-by-session interaction for each frequency band (top row), as well as edges showing a main effect of drug (middle row) and session (bottom row), as computed by RM-ANOVA. Connections showing significant interaction effects were observed in alpha (*n* = 83), beta (62), theta (61) and delta (7) bands, with most connections clustered around posterior regions (occipital / parietal). There were no edges in the gamma range demonstrating an interaction effect (Fig. S1 demonstrates only gamma^1^, 40 – 60 Hz, however the negative result was also observed in the higher gamma bands). Connections showing a main effect of drug were observed in theta (*n* = 96 edges), alpha (58), beta (33) and delta (20), largely confined to posterior regions but including interactions with cingulate and temporal regions in the alpha band. Effects of session were observed across all frequency bands (*n* = 130 edges in alpha, 111 in beta, 48 in theta, 22 in delta and 3 in gamma^1^). Again, these were posteriorly focussed but included interactions with frontal, deep (delta) and temporal (theta) regions. A list of the top-5 strongest (significant) edges for each frequency band, and each term (effect), are listed in supplementary Table 1.

**Fig. 2:**
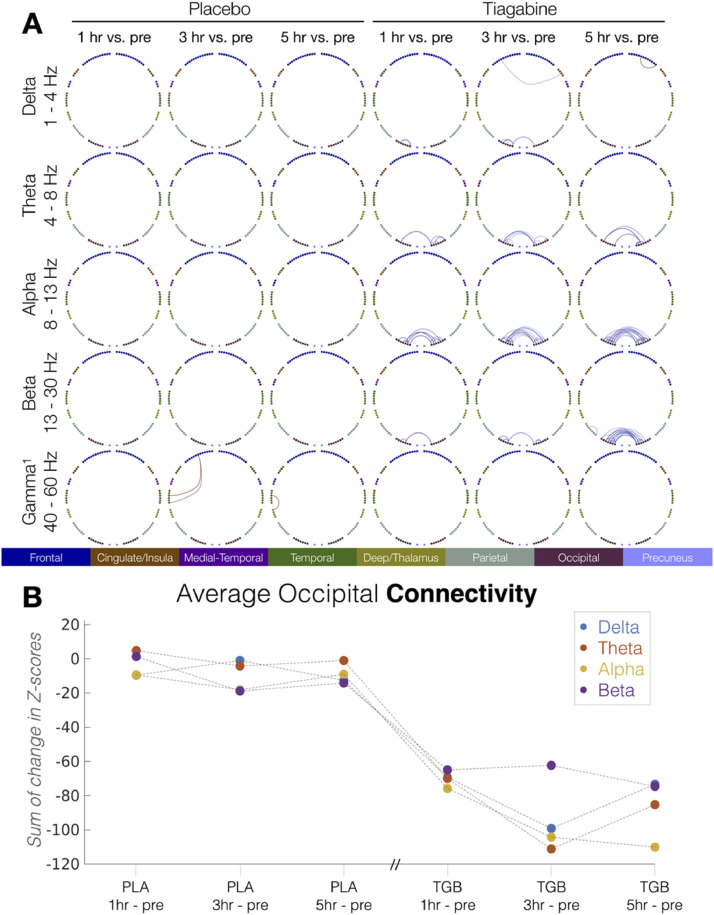
(A) Topographical representation of the post-hoc permutation-based (5k) tests, showing only significant (*p*<0.05) connections, for within-drug comparisons to baseline (1-, 3- and 5- hours vs. pre). Colour represents direction (red = increased vs. pre, blue = decreased vs. pre). (B) Time course of mean occipital connectivity effects for post-drug vs. baseline, for placebo (PLA) and tiagabine (TGB). Colours represent frequency bands. Note that the Placebo and Tiagabine sessions were performed on different days. (For interpretation of the references to colour in this figure legend, the reader is referred to the web version of this article.)

Post-hoc permutation-based randomisation testing of within-drug session effects revealed significant reductions (Fig. 2(A), blue lines) of edge strength for tiagabine at 1, 3 and 5 hr vs. pre, across delta, theta, alpha and beta. Analysis of the topography revealed these reductions were confined to occipital cortices. Increases were observed at two frontal edges (1 at 3 h, 1 at 5 h) in the delta band. Significant increases in edge strength were observed between frontal and temporal regions (3 h vs pre) and temporal and deep regions (5 h vs pre) across all three gamma windows for placebo. No other within-drug-vs.-pre changes in edge strength were observed for placebo. A list of all significant edges for each frequency band and each comparison (*n* = 6; 1, 3 and 5 h vs. pre for each drug) are listed in supplementary Table 2. Fig. 2(B) summarises the ‘time-course’ of effects on occipital connectivity for each post-drug session vs pre.

### 3.2 Source activity

**Fig. 3:**
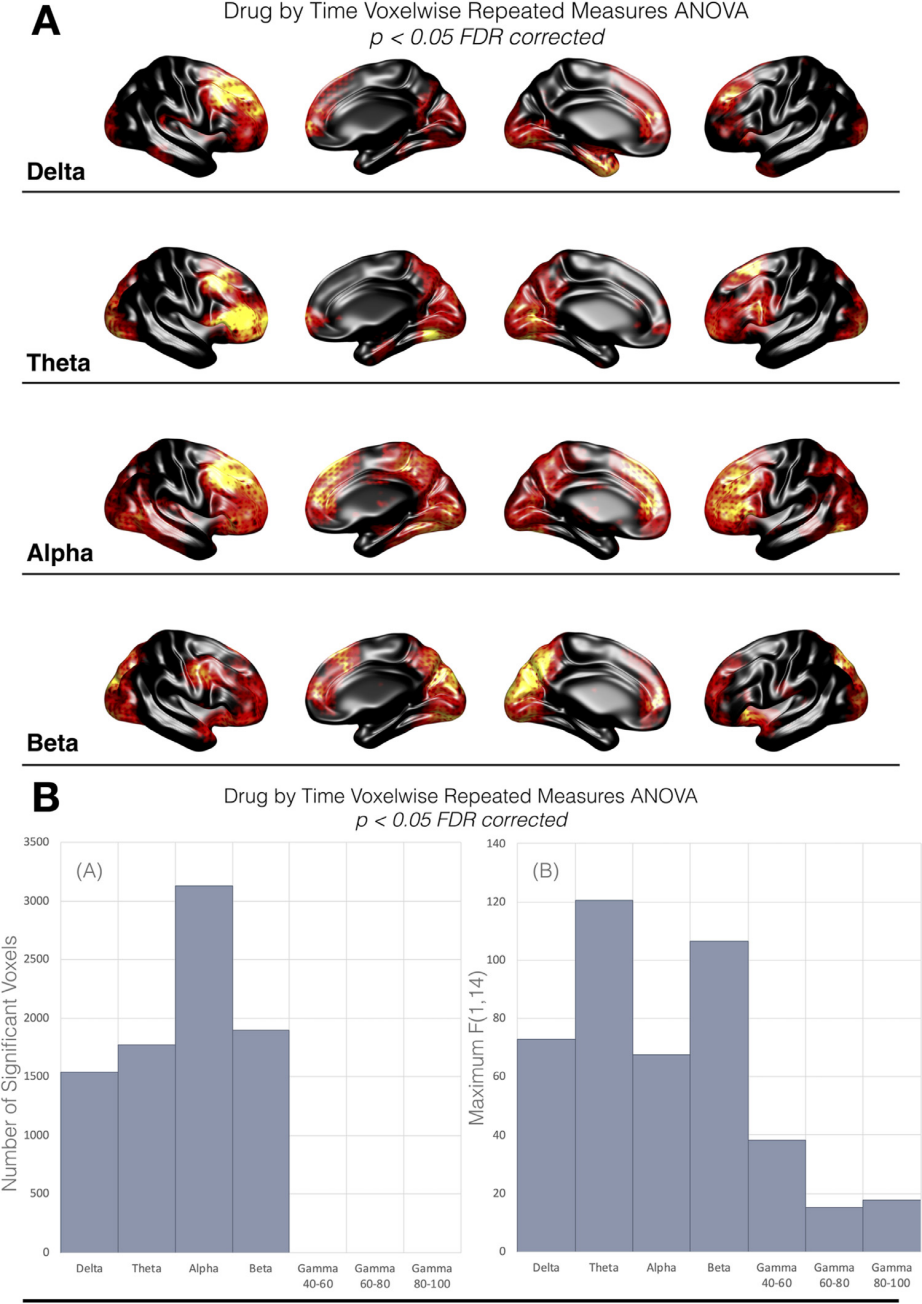
(A) Surface representation of the voxels demonstrating significant (*p*<0.05) drug-by-session interaction effects on source activity (from RM-ANOVA). Red-Yellow colour axis represents relative strength of F-statistic for significant voxels (*p*<0.05 FDR corrected). Black = *n*.s. (B) Summary plots for the voxelwise drug-by-session repeated-measures ANOVA, for source activity, showing (inset a) the number of significant voxels in each frequency band (*p* < 0.05 FDR corrected) and (inset b) the maximum F-statistic in each frequency band. (For interpretation of the references to colour in this figure legend, the reader is referred to the web version of this article.)

Fig. 3 shows the voxels whose ‘activity’ demonstrated significant drug-by-session interaction, for each frequency band (i.e. voxels with *p* < 0.05 after false recovery rate correction (FDR)) (Benjamini and Hochberg, 1995). Significant drug x session interaction effects on activity were observed in alpha (*n* = 3133 voxels), beta (*n* = 1900), theta (*n* = 1776) and delta (*n* = 1539, see Fig. 3(B)) but not gamma. Within each band, peak F-statistics in size order were F(1,14) = 120.37 (theta), F(1,14) = 106.43 (beta), F(1,14) = 72.87 (delta) and F(1,14) = 67.62 (alpha). These are summarised in Fig. 3(B).

**Fig. 4:**
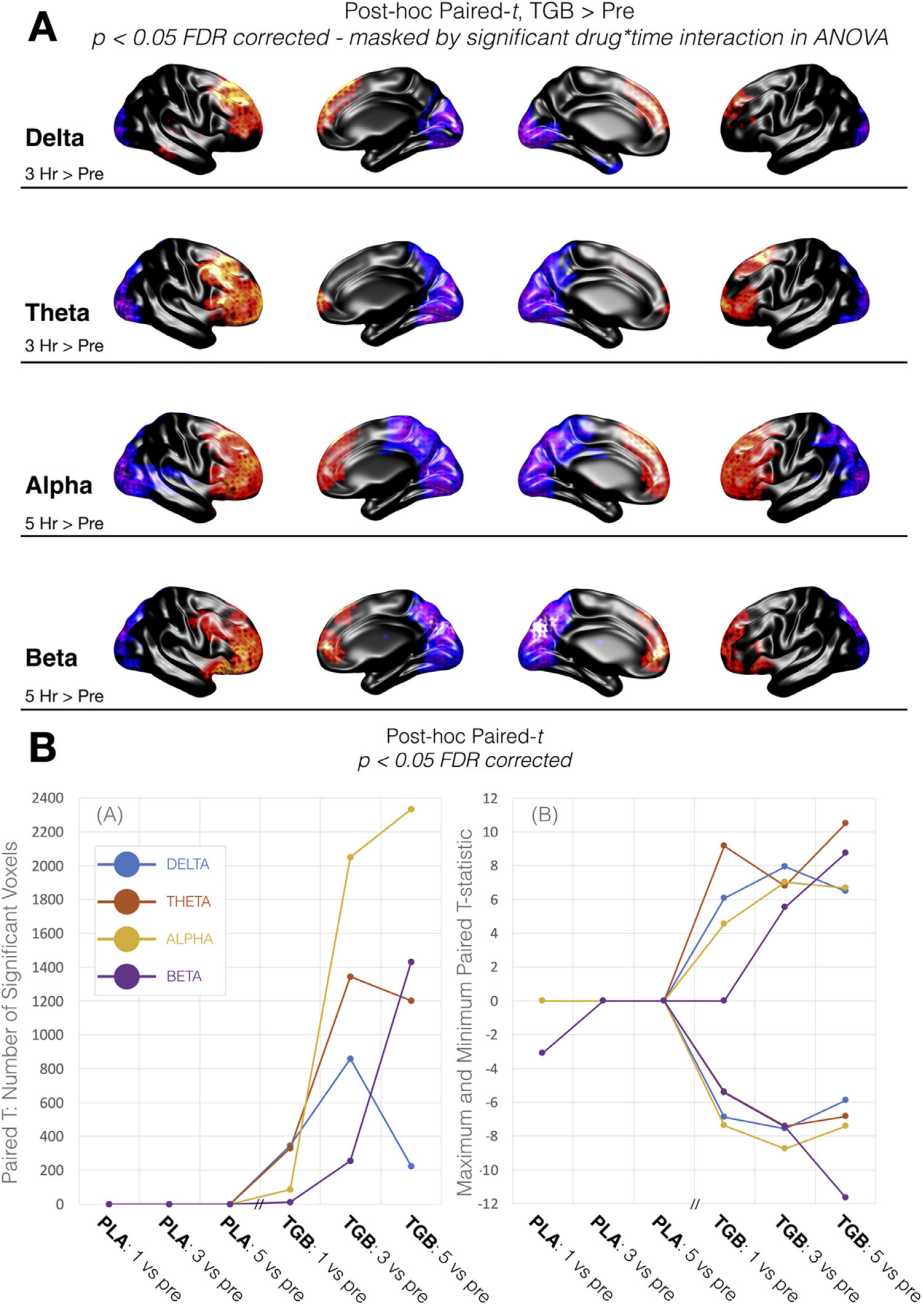
(A) Surface representation of the significant (*p* < 0.05 FDR corrected) post-hoc paired-t values for post-tiagabine vs pre, for activity measures in each frequency band. The post-drug session depicted was chosen based on the session showing the maximum number of significant voxels. For delta and theta, this was 3 hr post tiagabine vs pre, whereas for alpha and beta it was 5 hr post tiagabine vs pre. Colour axis represents direction of effect, with red/yellow representing significant, positive t-values, blue/purple representing significant negative t-values and black representing non-significant voxels. (B) Summary of the source activity post-hoc paired-t effects for post-drug vs pre for each session. (inset a) shows the number of significant (*p* < 0.05 FDR corrected) voxels for each frequency band. (inset b) shows the corresponding peak significant paired t-value for each comparison (x-axis) and frequency band. For clarity, the strongest positive and negative t-values are presented separately. Note that the Placebo (PLA) and Tiagabine (TGB) sessions were performed on different days. (For interpretation of the references to colour in this figure legend, the reader is referred to the web version of this article.)

Post-hoc paired-t tests between post-drug time points versus the pre-drug recording, were computed for voxels demonstrating significant interaction effects in the repeated measures ANOVA. The resulting t-difference maps for each frequency band, shown for tiagabine in Fig. 4(A), depict a pattern common across frequencies whereby frontal regions demonstrated increased activity and posterior regions decreased activity, in post-tiagabine sessions relative to pre-drug (this is shown only for tiagabine in Fig. 4, not placebo). Fig. 4(B) summarises the time-course of these paired-t effects over all sessions (all post-drug vs pre, for both tiagabine and placebo sessions).

### 3.3 Correlation between the spatial distribution of drug effects and mean flumazenil pet measures

**Fig. 5:**
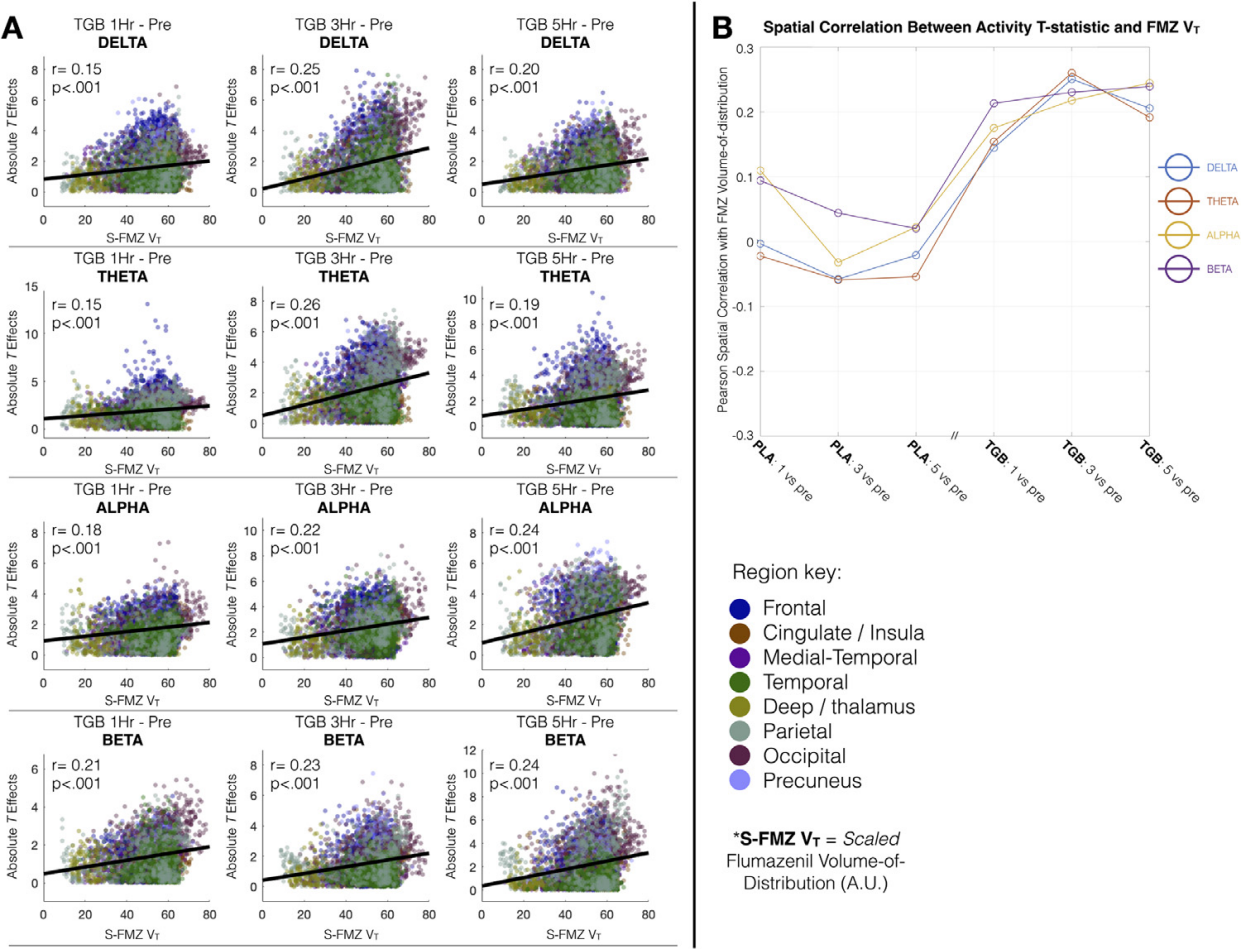
(A) Spatial distribution of scaled flumazenil FMZ VT correlations with effects of drug (absolute t value) on activity across frequency bands delta (top), theta, alpha and beta (bottom). Dots represent individual voxels, colour coded by AAL region to which they belong. Statistics (r and p-value) are derived from randomisation-corrected Pearson correlations with omnibus correction for multiple comparisons. Note that because of inherent spatial smoothing within both the PET and MEG beamformer maps, the p-values listed here are likely to be artificially small. (B) Summarising the spatial correlation of flumazenil volume-of-distribution with drug effects on activity across MEG sessions. Note that the Placebo (PLA) and Tiagabine (TGB) sessions were performed on different days.

We observed significant correlations (randomisation testing with 5000 permutations and omnibus correction) in the spatial distribution of the mean drug effects on activity (i.e. absolute paired-t value of drug vs pre) with scaled FMZ V_T_, across delta (*r* = 0.15, 0.25 and 0.2 at 1, 3 and 5 h, respectively), theta (*r* = 0.15, 0.26 and 0.19 at 1, 3 and 5 h, respectively), alpha (*r* = 0.18, 0.22 and 0.24 at 1, 3 and 5 h, respectively) and beta (*r* = 0.21, 0.23 and 0.24 at 1, 3 and 5 h, respectively). These correlations suggest that the magnitude of drug effects on activity, irrespective of sign, tended to be bigger in regions with higher FMZ V_T_ (Fig. 5).

## 4 Discussion

Our results indicate that pharmacologically increased GABA availability leads to reductions of both local activity and regional connectivity across low frequencies from delta, through to higher, beta frequencies. Connectivity effects are localised to posterior brain regions, with predominant effects in occipital lobe. Fig. 2(B) summarises the time course of mean effects for the connectivity, averaged across occipital regions, showing delta, theta, alpha and beta. Voxelwise analysis of ‘activity’ (temporal coefficient of variation) in each frequency band also demonstrated tiagabine induced reductions in posterior regions (Fig. 4), that were accompanied by increases in activity in frontal regions. Fig. 4(B) summarises the time course of activity effects for each frequency band.

As summarised in Fig. 2(B), the effects of tiagabine on functional connectivity across the four frequency bands show a similar temporal pattern, suggesting a common mechanism for the observed effects that is not frequency specific. This wide-band effect is consistent with theories of GABAergic function as a crucial component of oscillation generation, whereby inhibitory GABAergic currents exert temporal control over principal excitatory populations.

Using a ‘template’ map of flumazenil V_T_, which is a quantitative measure of flumazenil binding at GABA_A_ receptors, we demonstrated that the distribution of drug-induced effects in activity, as measured by the absolute paired-t value of post-drug vs pre, correlated with the distribution of GABA_A_ receptors across the brain (Fig. 5). In other words, regions with higher flumazenil binding also demonstrated bigger effects of drug on activity in delta, theta, alpha and beta bands. The ‘time course’ of this correlation across MEG session is summarised in Fig. 5(B). Interestingly, this time course suggests that the drug effects peak around the 5th hour scan, whereas self-reported subjective high, using the SHAS, peaks between the 1st and 3rd hours (supplementary Fig., S2), and had reduced by the 5th hour scan. While our limited sample size limits us from exploring this interesting discrepancy further in the present data, follow up studies with sufficient power to explore individual differences should explore this temporal dissociation between the peak in subjective effects and the peak in imaging metrics.

Previous studies have suggested that tiagabine is not directly active at GABA_A_ sites, instead exerting effects on the GABAergic system through inhibiting reuptake of GABA predominantly through GABA transporter 1 (GAT1) (Madsen et al., 2011; Ransom and Richerson, 2009) and the extrasynaptic betaine/GABA transporter BGT1 (Madsen et al., 2011).

Despite this lack of binding profile at GABA_A_ sites, tiagabine increases the decay time of GABA_A_ mediated inhibitory postsynaptic currents (IPSCs) (Thompson and Gähwiler, 1992) and increases the affinity of GABA_A_ receptors containing the benzodiazepine site to GABA (Frankle et al., 2009). This is important, since it links increased availability of GABA (through inhibited reuptake) to a functional effect or consequence, in the form of increased affinity and IPSC decay time. In other words, it demonstrates that merely increasing the amount of GABA with tiagabine does have a downstream effect on GABAergic function, via a GABA_A_ mediated phenomenon. As such, our findings of tiagabine-attenuated functional connectivity and activity at low frequencies across posterior cortex may reflect enhanced GABAergic function in the form of lengthened IPSCs. The correlation in spatial effects between activity and (template, canonical) FMZ V_T_ supports this, because the effects are spatially colocalised to regions with a higher density of GABA_A_ receptors.

The above argument cannot, however, explain the significant increases in activity observed in frontal regions after administration of tiagabine. Increased frontal power (not activity) of low frequency oscillations has been noted previously, albeit at the sensor level, with the GABA enhancing drugs tiagabine and gaboxodol, but not zolpidem (Nutt et al., 2015).

Inclusion of template flumazenil V_T_ data allowed us to go a step further than quantifying changes in connectivity with enhanced GABA, because we were able to demonstrate evidence that the amount of functional change in a region was predicted by that region's canonical density of GABA_A_ receptors. This provided a mechanistic link between the drug target and observed response, suggestive of target engagement. However, to make such an assertation follow-up studies to explore the specificity of this effect – both by exploring a PET map of a difference receptor, and by the inclusion of a non-GABAergic drug.

Although we observed connectivity and activity changes across frequency bands ranging from delta to beta, we observed no connectivity changes in the gamma band, even when sub-dividing into discrete 20 Hz windows. One explanation for this, is that the GABAergic processes altered by tiagabine did not disrupt the generative mechanisms of gamma oscillations. However, this is unlikely since the generation of (and spectral characteristics of) gamma oscillations are coupled to GABA_A_ dynamics (Bartos et al., 2007; Whittington et al., 2000). Instead, we propose that gamma oscillations, which are considered locally generated phenomena within cortical columns, are harder to measure between-region. This is because gamma oscillations, by virtue of being fast, can exist more transiently than lower frequencies, and are therefore harder to detect when correlating long amplitude envelopes. If this is the case, methods aimed at identifying fast dynamical switching, such as Hidden Markov Models, may be more successful for quantifying faster frequencies (Baker et al., 2014).

### 4.1 Limitations

This study used a template of flumazenil V_T_ derived from existing data, and from different subjects to those who took part in the tiagabine MEG study. Using averaged PET data as a canonical reference map of GABA_A_ density across the brain permitted us to compare to the average distribution of drug effects across the brain, but lacks the specificity afforded by having PET and MEG on the same individuals. Future studies should consider this, since having both would allow for detailed analysis of individual differences. A caveat here is the relative expense and exposure to radioactivity with PET compared with the safety of M/EEG. Future studies should also consider ‘control maps’ derived by PET ligands for other receptor types, which would allow exploration of the specificity of the effect to GABA-A receptor distribution.

A further limitation related to the use of MEG and PET data from different cohorts is the apparent age gap, where the participants in the PET template were on average 46, whereas the participants in the MEG were on average 25.5. This raises the question of age as a confound, since GABA receptor densities and levels may decline with age (Cuypers et al., 2018). However, in the case of age-related decline, our study actually made it harder to find a correlation between the two groups, since the older cohort do not have such a ‘dense’ map as the younger MEG subjects would have. Again, including the same participants in the MEG and PET portions of the study would eliminate this confound.

Our investigation of the spatial co-distribution of activity with FMZ V_T_ may overestimate correlations due to the inherent smoothness in the PET images. This smoothness-induced autocorrelation reduces the effective degrees of freedom, thereby rendering inflated statistics in conventional correlation measures, such as the Pearson's correlation employed here (Afyouni et al., 2019; Arbabshirani et al., 2014).

## 5 Conclusions

We have demonstrated that pharmacologically increased GABA availability led to increased frontal activity and reduced posterior activity – and inter-region connectivity – across multiple frequency bands. The spatially distinct pattern of activity changes correlated with a template distribution of GABA_A_ receptors across the brain. We propose a mechanistic explanation for our results, whereby increased GABA availability (by tiagabine) led to increased affinity of GABA_A_ receptors for GABA (Frankle et al., 2009), lengthening IPSCs (Thompson and Gähwiler, 1992) and consequently reducing low frequency oscillatory activity and connectivity (by the lengthened IPSCs ‘dampening’ current fluctuations underpinning macroscopic oscillations). While demonstrating a role for GABAergic dynamics in shaping the activity and topography of functional connectivity, our results clearly indicate the need for follow-up studies to assess the potential utility of MEG-based measures of connectivity and activity as tools for time and frequency-resolved electrophysiological receptor mapping.

## Role of the funding source

The work presented here was supported by CUBRIC and the School of Psychology at Cardiff University as well as an MRC UK MEG Partnership Grant, MR/K005464/1. ADS & HLC are supported by a Wellcome Strategic Award (104943/Z/14/Z).

AH acknowledges support by the Wellcome EPSRC Centre for Medical Engineering at King's College London (WT 203148/Z/16/Z) and the Department of Health via the National Institute for Health Research (NIHR) comprehensive Biomedical Research Centre award to Guy's & St Thomas’ NHS Foundation Trust in partnership with King's College London and King's College Hospital NHS Foundation Trust. Data for the FMZ template were originally acquired at the MRC Clinical Sciences Centre, Hammersmith Hospital.

## Author contributions

Study conception & hypothesis: ADS & KDS.

Data collection: KH, SDM & AH.

Data analysis & statistics: ADS, HLC, KH, SDM, AH & KDS.

Drafting manuscript: ADS, HLC & KDS.

Manuscript revisions: ADS, HCL, KH, SDM, AH & KDS.

## Declaration of Competing Interest

All authors declare no conflict of interest.

## Appendix Supplementary materials

Image, image 1

Image, image 2

Image, application 3
