# Supplementary figures and images for: Tiagabine induced modulation of oscillatory connectivity and activity match PET-derived, canonical GABA-A receptor distributions
Source: Eur Neuropsychopharmacol. 2021 Sep;50:34–45. doi: 10.1016/j.euroneuro.2021.04.005 (PMC8415204; doi:10.1016/j.euroneuro.2021.04.005)

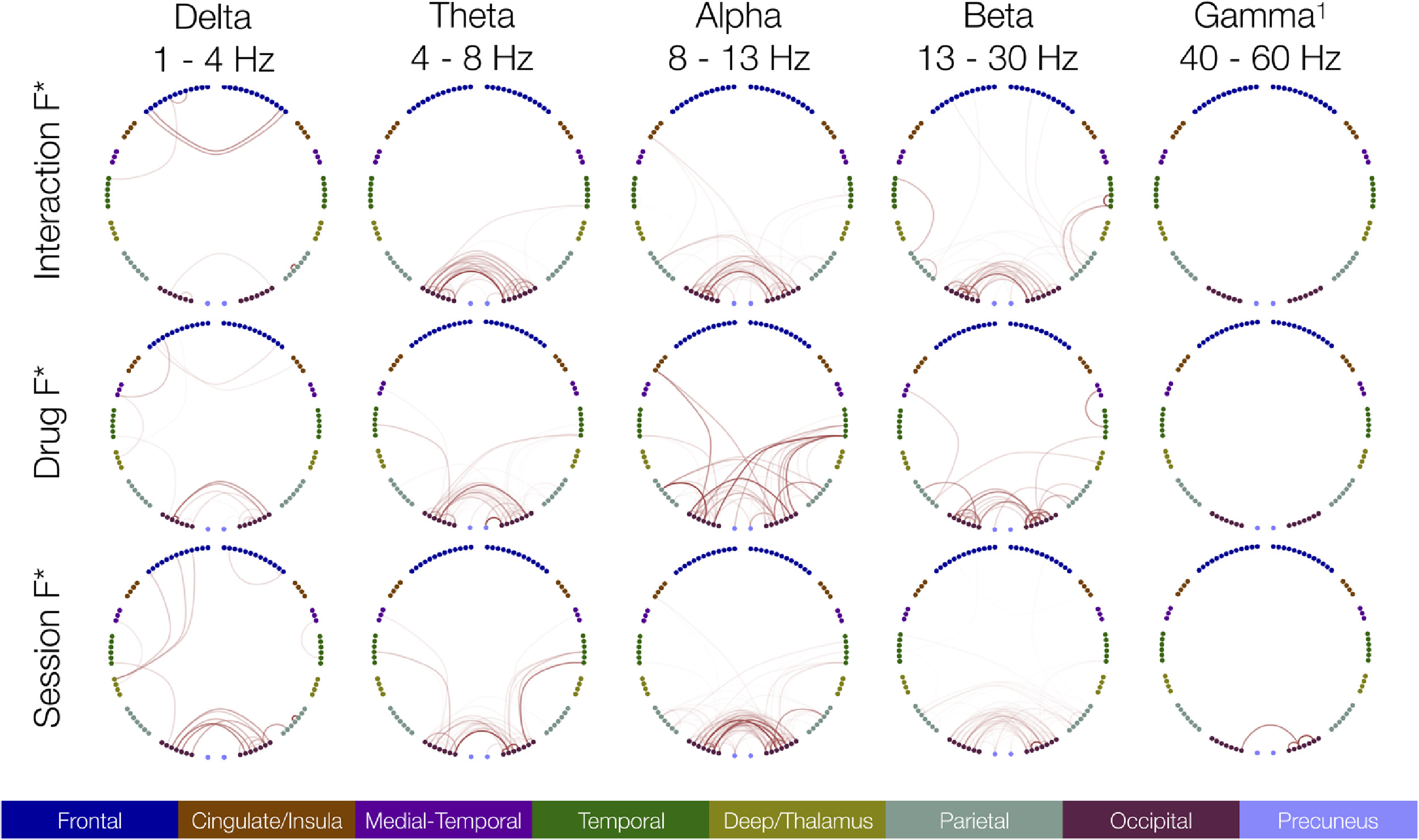

Supplement: Supplementary file 2 [file mmc2.jpg]

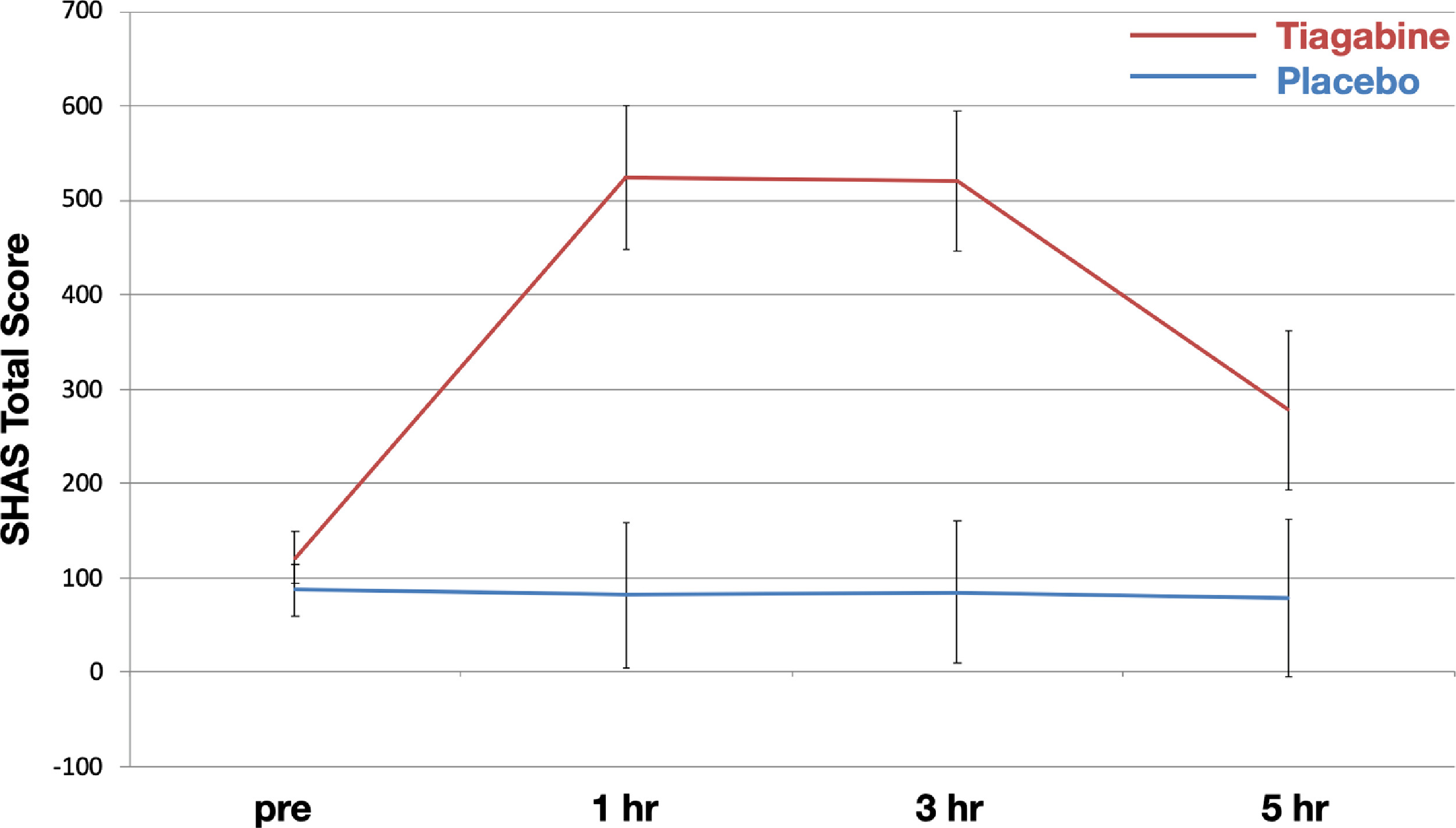

Supplement: Supplementary file 3 [file mmc3.jpg]
